# Perspectives on the Use of Stem Cells for Autism Treatment

**DOI:** 10.1155/2013/262438

**Published:** 2013-10-10

**Authors:** Dario Siniscalco, James Jeffrey Bradstreet, Nataliia Sych, Nicola Antonucci

**Affiliations:** ^1^Department of Experimental Medicine, Second University of Naples, Via S. Maria di Costantinopoli, 16-80138 Napoli, Italy; ^2^Centre for Autism, La Forza del Silenzio, 81036 Caserta, Italy; ^3^Cancellautismo, 50132 Florence, Italy; ^4^International Child Development Resource Center, Chateau Élan, Braselton, GA 30517, USA; ^5^Clinical Department, Cell Therapy Center EmCell, Kiev 04073, Ukraine; ^6^Biomedical Centre for Autism Research and Treatment, 70126 Bari, Italy

## Abstract

Autism and autism spectrum disorders (ASDs) are complex neurodevelopmental disorders. ASDs are clinically defined by deficits in communication, social skills, and repetitive and/or restrictive interests and behaviours. With the prevalence rates for ASDs rapidly increasing, the need for effective therapies for autism is a priority for biomedical research. Currently available medications do not target the core symptoms, can have markedly adverse side-effects, and are mainly palliative for negative behaviours. The development of molecular and regenerative interventions is progressing rapidly, and medicine holds great expectations for stem cell therapies. Cells could be designed to target the observed molecular mechanisms of ASDs, that is, abnormal neurotransmitter regulation, activated microglia, mitochondrial dysfunction, blood-brain barrier disruptions, and chronic intestinal inflammation. Presently, the paracrine, secretome, and immunomodulatory effects of stem cells would appear to be the likely mechanisms of application for ASD therapeutics. This review will focus on the potential use of the various types of stem cells: embryonic, induced pluripotential, fetal, and adult stem cells as targets for ASD therapeutics.

## 1. Introduction

### 1.1. Autism Spectrum Disorders: Overview

According to the Diagnostic and Statistical Manual of Mental Disorders (DSM-IV) published by the American Psychiatric Association, autism spectrum disorders (ASDs) are complex, severe, heterogeneous neurodevelopmental disorders [1]. The core characteristics of ASDs are dysfunctions in social interaction and communication skills, restricted interests, repetitive and stereotypic verbal and nonverbal behaviours [2, 3]. Several biochemical and cellular events are associated with ASDs: oxidative stress, endoplasmic reticulum stress, decreased methylation capacity, limited production of glutathione, mitochondrial dysfunction, intestinal dysbiosis and inflammation, increased toxic metal burden, impaired detoxification, and dysregulation of the brain's intrinsic immune system (including autoimmunity and activation of neuroglial cells) [4, 5]. Despite this extensive body of evidence for an underlying immunotoxicological event in the development of autism, the exact origins of pathogenesis and pathophysiology of ASDs remain to be fully elucidated.

Equally, the presently approved pharmacotherapy fails to address the documented biological aberrations and rather only targets specific behavioural symptoms (i.e., agitation or hyperactivity). Other available treatments for ASDs can be divided into behavioural, nutritional, and biomedical approaches, but a defined standard approach has not been generally accepted [6]. With few well-controlled clinical trials, clinicians are left with the complex challenges of crafting individualized interventions based on limited biomarkers [4, 7, 8]. Novel findings of epigenetic, neuroimmunologic, and environmental changes observed in ASDs suggest that stem cell therapies could be potential interventions for treating autistic syndromes, while enhancing early interventions for autism management in the future [9].

## 2. Stem Cells in Autism Spectrum Disorders

It is generally agreed that stem cell therapies represent the future of molecular and regenerative medicine for what would otherwise be untreatable human diseases. Stem cells are also suitable for developing cell-based patient-specific pharmacotherapies [10, 11]. Thus, it is hoped that stem cells offer new treatment options for ASDs [12]. The immune and neural dysregulations observed in ASDs provide specific targets for stem cell therapies. Stem cells possess several useful characteristics which suggests there potential therapeutic application for ASDs. These are (1) their self-renewal ability: stem cells are able to generate more identical stem cells; (2) differentiation process: through it, the cells give rise to more differentiated cells; (3) paracrine regulatory functions: stem cells synthesize and release a complex and implantable “biopharmacy,” capable of regulating cell differentiation, tissue and organ repair, and anti-inflammatory actions in the recipient. The cell type and programming of the stem cell determines its potential paracrine pharmacology. The biopharmacy of stem cells: trophic and immunomodulatory human-specific biomolecules may equally be isolated in the laboratory and used as specific therapeutic agents without the actual implantation of the cells into the patient.

These paracrine functions of stem cells (i.e., the biopharmacy or the secretome) are attracting much consideration [13, 14]. It has been already proposed that in ASD cell-based treatment, the positive effects that could be mediated by stem cells could be achieved through the trophic and immunomodulatory properties [9]. Implanted stem cells (whether autologous or donor) are able to affect the recipient immune system through two proposed mechanisms: (1) cell-to-cell contact activation mechanism, through which transplanted stem cells switch proinflammatory macrophages to anti-inflammatory macrophages [15, 16]; (2) paracrine-secretome activity [17]. It is noteworthy to consider that ASDs are associated with significant immune alterations and proinflammatory cytokines overproduction [18]. We propose that through these mechanisms stem cells could simultaneously counterbalance the immune system aberrations, while activating endogenous restorative mechanisms within damaged tissues contributing to recovery of functional deficits. Recipient cell replacement by transplanted stem cells, while potentially beneficial, is not therefore a necessary prerequisite for effective stem cell therapeutics since these other mechanisms may be sufficiently restorative.

This review will focalize on the major types of stem cells, embryonic, fetal, and the adult stem cells, that could offer specific advantages in cell transplantation for ASD treatment.

## 3. Embryonic Stem Cells

Apart from their ethical controversies, embryonic stem cells (ESCs) are pluripotent stem cells derived from early stages of embryonic development [19–21]. ESCs are obtained from the inner cell mass of the blastocyst-stage preimplantation embryo, single blastomeres of the morula stage. Their pluripotency capacity means the capability of differentiating into all three potential germ layers: ectoderm, mesoderm, and endoderm. Proper regulation of posttransplantation ESCs is the daunting challenge. They derive their cell differentiation characteristics from the recipient environment. However, absent antirejection medication would be expected to be rejected by the recipient immune system once HLA-II expression occurred [22]. The challenges of chimeric engrafting are far from understood in the pediatric population, and thus this potential outcome from ESCs is poorly characterized in the scientific literature and a source of concern. While still at the embryonic stage of their development, they are, however, considered potent producers of paracrine activity [23].

In ASDs, it has been demonstrated that an altered immune cell ratio is sometimes associated with a decreased number of T lymphocytes [18]. The ability of ESCs to differentiate into hematopoietic cell lineages, giving rise to all blood cell types and subtypes of the immune system (i.e., T cells, NK cells, and dendritic cells), could be used in immune-altered pathologies, such as ASDs, which require induction of the immune response in an antigen-specific manner [24–26]. Some concerns on the use of ESCs have been raised. ESC safety, efficacy, and long-term benefits have thus far been suboptimal [27]. *In vivo* teratoma formation seen after implantation of undifferentiated ESCs and their uncontrollable cell proliferation has provided some critical issues precluding clinical transfer of ESC transplantation [28–30]. 

To date, there is evidence of just one phase I clinical trial conducted with ESCs as compared to the numerous trials conducted with adult stem cells [31].

Recently, the King's College London has deposited pedigreed human ESCs (hESCs) potentially pure enough to be used in therapies into the UK Stem Cell Bank. These hESCs are considered high quality and “clinical-grade” cells produced under certified manufacturing conditions without using any of the animal cells or products typically needed [32]. However, it could take several years before the cells make their way into humans, as further experiments, in order to check eventually genetic abnormalities or other problems that would prevent their use in therapies need to be performed. Moreover, while some years ago, the Singapore-based biotech company ESI launched four safe embryonic stem-cell lines specifically manufactured for clinical use [33], recently, the biotechnology company Geron, which was the first company to be approved by the US Food and Drug Administration (FDA) to test ESCs in a human trial, announced that the trial was stopped and cited cost issues with the study [34].

Based on these issues, it is doubtful whether ESCs are ready for clinical therapy. At present, we therefore consider it intriguing to consider their *in vitro* secretome functions when designing restorative pharmacological cocktails for autism, as opposed to their transplantation potential. This functional use of ESCs as biolaboratories to produce specific secretome activity would lend itself to animal modeling and facilitate the ultimate progression to potential human applications.

Equal in posttransplantation behavior and differentiation is the category of induced pluripotential stem cells (iPSCs). With this population of stem cells, more mature cells, such as mesenchymal stem cells, are induced through genetic manipulation to convert to pluripotential activity. The long-term effects of the iPSCs alterations on posttransplantation safety and activity are far from developed [35]. So again, we would propose that for application to pediatric populations with ASDs, these cells are still best suited for *in vitro* development of secretome-derived pharmaceuticals.

## 4. Fetal Stem Cells

Fetal stem cells (FSCs) are a subpopulation resident within fetal tissues. Fetal-derived tissues typically contain committed and differentiated cells in addition to the FSCs. These fetal tissues and their associated FSCs divide into 3 subtypes: ectodermal (including brain), mesodermal, and endodermal. They have great potential for clinical use; as they possess immune-regulatory functions as found in mesenchymal stem cells but exhibit a greater expansion capacity and enhanced plasticity [36]. Indeed, FSCs are more rapidly, easily, and efficiently reprogrammed to pluripotency than neonatal and adult cells [37]. Early gestational fetal neuronal tissue is of particular interest to neurodegenerative disease therapies and may serve as a model for ASD interventions. In part, this is because early FSCs have minimal or no expression of MHC-I and no MHC-II [38]. Further, FSC-derived hematopoietic cells express HLA-G a factor in tolerance, thereby conferring increased viability posttransplantation [38]. These factors may contribute to the success of allogeneic FSC transplants in ASD therapies.

It has been reported that fetal mesenchymal stem cells exert strong immunomodulatory effects [39], possess a stable phenotype, and demonstrate less senescence. In addition, unlike ESCs, they are not able to form teratomas posttransplantation and are obtained from tissues that would otherwise be discarded [40]. In the first phase of their life and unlike MSCs, FSCs express baseline levels of the pluripotency stem cell markers Oct-4, Nanog, Rex-1, SSEA-3, SSEA-4, Tra-1-60, and Tra-1-81, whereas in the second phase they express mesenchymal stem cell markers, that is, CD73, CD90, and CD105, and are not able to express haematopoietic or endothelial markers (i.e., CD14, CD34, and CD45) [41].

Fetal tissue transplantation has become a potential symptomatic treatment and disease management option for patients suffering from neurodegenerative diseases [42]. Cells isolated from first trimester human fetuses have the capacity to survive after transplantation, acquire a mature neuronal phenotype, and mediate a functional effect [42]. FSC benefits could be due to paracrine trophic actions on host tissues (particularly, immune, brain and gastrointestinal tissues), rather than cell replacement. FSCs are able to produce and release several diffusible neurotrophic and growth factors [43, 44]. Their capacity to suppress proinflammatory cytokines is another key mechanism of action of possible application to ASD therapeutics [45].

As FSCs are derived from ectodermal, mesodermal, and endodermal layers, they retain their tissue-specific instructions and are therefore regulated properly, unlike pluripotential ESCs. In this way, cell or tissue/organ-specific FSCs could restore dysfunctional development of the brain, gut, and immune system. FSCs also lend themselves to readily available animal models of autism which may further ease their preclinical laboratory investigations.

## 5. Adult Stem Cell Types

### 5.1. Mesenchymal Stem Cells

Mesenchymal stem cells (MSCs) transplantation or autologous reimplantation could be a useful therapeutic tool in the future of regenerative medicine [46–48]. They are self-renewing precursor cells of mesodermal origin found principally in the bone marrow and adipose of children and adults, which can differentiate into bone, fat, cartilage, and stromal cells of the bone marrow [49–51]. Interestingly, MSCs are able to differentiate into both mesenchymal and nonmesenchymal lineages, as these cells retain multilineage potential. According to the work of Dominici and to The International Society of Cellular Therapy (http://www.celltherapysociety.org/), MSCs are defined by the following minimal set of criteria: (1) capacity to grow in adherence to plastic surface of dishes when maintained in standard culture conditions; (2) to express cytospecific cell surface markers, that is, CD105, CD90, and CD73, to be negative for other cell surface markers, that is, CD45, CD34, CD14, and CD11b; (3) capacity to differentiate into mesenchymal lineages, under appropriate *in vitro* conditions [52, 53]. However, further review on their definition and origin is needed in the light of recent observations [54, 55]. MSCs possess high expansion potential, genetic and phenotypic stability, high proliferation rate as cultured-adherent cells, and self-renewal capacity [56].

These cells are considered very useful for transplantation purposes, as they are clinically safe, show immune-modulating capabilities, are easily obtained from pediatric and adult tissues, and quickly expanded as well as stored. In addition, once transplanted, MSCs are able to migrate and to home to the sites of tissue injury. MSCs have strong anti-inflammatory and immunosuppressive activity, rendering them very attractive for successful autologous, as well as heterologous, transplantations without requiring pharmacological immunosuppression [57, 58]. They do not give uncontrollable growth or tumour formation [59]. There is no need of genetic modification or pretreatment before transplantation; since MSCs are able to express *in vivo* immunosuppressive factors, immune rejection problems are overcame [60, 61]. Of course, moral objections or ethical issues are not due to MSCs [62].

We already discussed on how MSCs could be useful for ASD therapy [9]. Briefly, MSCs could stimulate the plastic response in the host damaged tissue, synthesize and secrete survival-promoting growth factors, restore synaptic transmitter release by providing local reinnervations, integrate into existing neural and synaptic network, and restoring plasticity [27]. The paracrine action of MSCs seems to be the most plausible and reasonable mechanism for the functional benefit derived from MSC transplantation. Indeed, MSCs are able to produce a large array of trophic and growth [63]; these biofactors could activate endogenous restorative mechanisms within injured tissues contributing to recovery of function lost as a result of lesions [27, 64].

In ASD therapy, MSC-mediated immune system modulating activity could be a key mechanism. As stated above, MSCs have a strong long-lasting immunosuppressive activity mediated via soluble biofactors. Through this activity, MSCs modulate the immune system. Indeed, it has been demonstrated that MSCs are able to inhibit the proliferation of CD8+ and CD4+ T lymphocytes and natural killer (NK) cells, suppress the immunoglobulin production by plasma cells, inhibit the maturation of dendritic cells (DCs) while not inhibiting regulatory T cells, and downregulate the T lymphocyte proinflammatory cytokine production [65–68].

Immune system dysregulation has been shown in ASDs, together with innate and adaptive altered responses [69–71]. ASD children show imbalances in CD3+, CD4+, and CD8+ T cells, as well as in NK cells. In addition, peripheral blood mononuclear cells (PBMCs) extracted from ASD children show overproduction of caspase proteases, proinflammatory cytokines, and cannabinoid-type 2 receptor, probably resulting in long-term immune alterations and proinflammatory cellular events [18, 72, 73]. It is noteworthy to consider that the MSC immunoregulatory effects could restore the immune imbalance in ASDs. Indeed, MSCs strongly inhibit TNF-*α* and INF-*γ* production and increase IL-10 levels resulting in inhibition of T-cell recognition and expansion [66]. Beyond T cells, MSCs are able to affect also other cells of the immune system (i.e., NK cells). This characteristic of MSCs is mediated through secretion of large amounts of several bioactive molecules (paracrine activity), that is, PGE-2 and IL-10. These molecules affect the T-cell mediated responses [18].

MSCs could also restore the defective cortical organization, plasticity dysregulation, and the injured brain functioning of ASDs. Abnormal functioning and cerebellum alterations have been identified in postmortem brains from ASD patients [74–77]. Indeed, supporting this potential application of cell therapies for ASDs, transplanted MSCs are able to promote synaptic plasticity and functional recovery [78, 79]. The ability of MSCs to migrate to the sites of injury where they can act in the repair process after implantation is another proposed mechanism of action of brain restoration and tissue repair played by MSCs [80].

ASDs are now recognized as epigenetic disorders [81]. Native MSCs in ASDs could not inhibit the epigenetic processes triggered by the environmental factors which ultimately led to the development of the autistic phenotype. Whether or not autologous MSCs are capable of responding appropriately to induce the healing effects needed for neuro-rehabilitation remains to be further determined.

### 5.2. Specific Mesenchymal Stem Cell Subtypes

#### 5.2.1. Adipose-Derived Mesenchymal Stem Cells

Adipose-derived mesenchymal stem cells (AD-MSCs) are abundant in human adipose [82]. They are readily harvested with minimally invasive procedures (e.g., small volume lipo-aspirate). These cells gained much consideration for autologous cell therapy. AD-MSCs show anti-inflammatory characteristics and are able to differentiate into adipogenic, osteogenic, chondrogenic, and other mesenchymal lineage [83]. Even though several clinical trials have been conducted using AD-MSCs, considerable uncertainty about their real clinical potential is still present. Their differentiation processes into cell lineages apart from adipocytes have not yet been definitively demonstrated in humans post-reimplantation. There is equally no evidence that the re-administration of MSCs extracted from adipose tissue will overcome the intrinsic sense of the MSCs to return to the surgical harvest (lipo-aspirate) site to initiate repair. Concerns on purity and molecular phenotype for AD-MSCs have also been raised. It is likely that cell preparations contain heterogeneous populations of cells. This fact creates uncertainty over whether AD-MSCs themselves are responsible for observed effects [83]. While AD-MSCs raise few significant safety concerns, further investigations on AD-MSC biology are needed before their use in ASD therapy can be further understood.

#### 5.2.2. Umbilical Cord-Derived MSCs

Other sources of stem cells include both umbilical cord and placenta [84]. These are abundant sources of stem cells, since the majority of post-delivery umbilical cords and placentas are discarded. Umbilical cord blood-derived mesenchymal stem cells are hematopoietic and have potential applications along those lines as previously discussed. The stroma of the cord is also a source of relatively primitive stem cells residing in the Wharton's jelly (WJCs). Their potential application resides in their low immunogenicity. WJCs express low levels of human leukocyte antigen (HLA)-ABC and no HLA-DR. WJCs are able to inhibit the proliferation of phytohemagglutinin-stimulated human peripheral blood lymphocytes and mouse splenocytes [85]. WJCs show also immunomodulation properties and act as trophic support to neighbouring cell populations [86]. These cells have potential for future applications in CNS regenerative medicine, as well as in ASDs. Equally, their *in vitro* capacity to produce paracrine trophic factors should be explored.

## 6. Neural Stem Cells

Another promising stem cell type for treating nervous system diseases are specific neural stem cells (NSCs) or neuroprogenitor cells (NPCs) [87]. NSCs can be isolated from both fetal and adult stem cells. These multipotent cells show self-renewal capacity and are able to generate multiple cell types of the mammalian central nervous system [88]. The capacity of integration into neural tissue, replacing damaged cells and reconstructing neural circuitry are the main characteristics of NSCs with potential usefulness for treating ASDs. In autism, excitatory and inhibitory cortical neurons contribute to minicolumn structure abnormalities and functional imbalance in cortex [88]. Small subsets of ASD patients show impairments in several synaptic-related genes [89]. Transplanted NPCs could promote neural tissue repair through their local contribution to changes in the brain microenvironment [87, 90]. Before being suitable for clinical applications in neurodegenerative diseases or ASDs, some critical issues with the use of NPCs require further investigation. A reliable source of sufficient autologous NPCs needs to be identified. Further, the regulation of postimplantation neural plasticity and differentiation of NSCs in the child or adult nervous system must be further defined [87].

## 7. Conclusions

Cellular therapies offer a needed and novel treatment modality in ASDs. Several stem cell types could be suitable for ASD therapy. Among them, MSCs and FSCs seem to show several biological advantages. However, long-term safety of cell-based therapies is not yet well established, and preclinical animal models are urgently needed to progress this area of research.
